# Personal

**Published:** 1895-12

**Authors:** 


### Personal.

Hon. James O. Putnam, of Buffalo, who has been a member of the council of the University of Buffalo since its foundation, fifty years ago, has been elected chancellor, to fill the vacancy caused by the death of the Hon. E. Carlton Sprague. This is a deserved though tardy recognition of the talent and services of Mr. Putnam and is, no doubt, a greater gratification to his friends than to himself. Hon. Wilson S. Bissell was chosen vice-chancellor upon the promotion of Mr. Putnam.

---

Dr. X. O. Werder, of Pittsburg, (*Pittsburg Medical Review*,) one of the original members of the editorial staff of the *Review*, has recently been elected to fill the chair of professor of diseases of women in the Western Pennsylvania Medical College. Dr. Werder takes the place of Professor W. J. Asdale, who recently resigned his position as a member of the faculty. Dr. Werder's clear judgment, superior abilities of diagnosis and operative skill make him an exceedingly valuable addition to the teaching staff of the college, and we predict for him a measure of success second to none.

Dr. J. Henry Dowd, of Buffalo, has gone to New York and Philadelphia, where he will spend a few weeks in post-graduate medical schools in preparation for the practice of a specialty.

Dr. Eli H. Long, of Buffalo, has been appointed a member of the governing council of the Dental Department of Buffalo University.

Dr. Lillian C. Randall, of Buffalo, who established the Riverside hospital in 1892, has removed it to 327 Breckenridge street.

Dr. John H. Daniels, of Buffalo, has been appointed lecturer on anatomy in the Medical Department of Niagara University.

